# Factors Associated With Risky Drinking Decisions in a Virtual Reality Alcohol Prevention Simulation: Structural Equation Model

**DOI:** 10.2196/56188

**Published:** 2024-05-06

**Authors:** Robert Hrynyschyn, Julie Dalgaard Guldager, Daniel Schulze, Patricia Bianca Lyk, Gunver Majgaard, Christiane Stock

**Affiliations:** 1 Charité – Universitätsmedizin Berlin, Corporate Member of Freie Universität Berlin and Humboldt-Universität zu Berlin, Institute of Health and Nursing Science Berlin Germany; 2 Leibniz ScienceCampus Bremen Digital Public Health Bremen Germany; 3 Unit for Health Promotion Research Department of Public Health University of Southern Denmark Esbjerg Denmark; 4 Research Department University College South Denmark Haderslev Denmark; 5 Charité – Universitätsmedizin Berlin, Corporate Member of Freie Universität Berlin and Humboldt-Universität zu Berlin, Institute of Biometry and Clinical Epidemiology Berlin Germany; 6 The Maersk Mc-Kinney Moller Institute Game Development and Learning Technology University of Southern Denmark Odense Denmark

**Keywords:** alcohol, prevention, virtual reality, risk behavior, structural equation model

## Abstract

**Background:**

Risky alcohol consumption among adolescents is a significant public health concern in most Western countries. Various motives and factors (eg, sensation seeking, gender, reduced self-efficacy) known in the literature are associated with risky drinking decisions in real life. Efforts to tackle risky drinking decisions in real life through skills training to deal with social pressures have been successful. However, interventions of this nature require significant resources. Technological solutions, such as virtual reality (VR), offer advantages, as they enable immersive experiences that replicate real-life scenarios. However, a question persists pertaining to the fidelity of real-world behaviors within virtual environments.

**Objective:**

This study is exploratory and aims to ascertain if the established drinking motives and factors for risky drinking decisions are transferrable to the virtual environment in the simulation game VR FestLab and to uncover determinants linked to risky drinking decisions within the simulation.

**Methods:**

The study analyzed data from the intervention arm of a cluster-randomized study of 161 Danish students aged 14-18 years who tested the virtual alcohol prevention simulation VR FestLab. At baseline and before playing VR FestLab, independent variables such as age, gender, alcohol consumption, use of other drugs, sensation seeking, drinking refusal skills, knowledge of blood alcohol concentration, and refusal communication skills were recorded. The dependent variable, virtual risk decisions, was measured immediately after the gameplay. Confirmatory factor analysis and structural equation modeling were used to examine the latent variables in relation to virtual risk decisions. Moderation analyses for age and gender in relation to the latent characteristics and the primary outcome were also conducted.

**Results:**

The data indicate that 73.9% (119/161) of the participants engaged in binge drinking at least once in their lifetime. The confirmatory factor analysis demonstrated a good fit of the items for their respective constructs; therefore, they were adopted without modification in the structural equation model. The data suggest that individuals with prior alcohol experience are 4 times more likely to engage in virtual risk decisions within the simulated environment (odds ratio 4.31, 95% CI 1.70-10.84; *P*=.01). Knowledge and awareness of blood alcohol concentration were associated with a lower chance to engage in virtual risk decisions (odds ratio 0.32, 95% CI 0.11-0.93; *P*=.04)*.* However, no significant associations were found between virtual risk decisions and other latent variables. Gender and age did not moderate the associations.

**Conclusions:**

The immersive and lifelike properties of VR partially reflected risk-related decisions. However, it remains unclear which factors favor the mapping of real-world behaviors in virtual simulations. Therefore, future research should address the mechanisms underlying behavioral dynamics in virtual simulations and explore the translation of virtual behaviors into real behaviors to gain a comprehensive understanding of the potential of virtual simulations for alcohol prevention.

## Introduction

Alcohol prevention continues to represent a pertinent public health concern worldwide [1]. Despite witnessing a reduction on a global scale since the establishment of the Millennium Development Goals in 1995, alcohol consumption still contributes to 5% of disability-adjusted life years and 5% of total fatalities [2]. Furthermore, harmful alcohol consumption is pivotal in over 200 diseases and injury conditions [3]. Notably, alcohol prevention remains significant, particularly among adolescents, as indicated by the latest European School Survey Project on Alcohol and Other Drugs report, which reveals that approximately 80% of students aged 15-16 years have experimented with alcohol at least once [4]. Denmark surpasses the European average, with roughly 92% of adolescents having consumed alcohol at least once in their lifetimes [4].

To effectively deter premature substance use among adolescents, targeted alcohol prevention programs prove indispensable [5]. In this context, it is important to consider the motives underlying risky consumption patterns among adolescents. A systematic review conducted by Adan et al [6] revealed an association between risky drinking behavior and specific personality traits. For instance, binge drinking was correlated with increased impulsivity and sensation seeking [6], while Stautz and Cooper [7] and Percy et al [8] established an association between high sensation seeking and an increased likelihood of heavy episodic drinking. Other studies have identified binge drinking as particularly linked to male gender and reduced self-efficacy [9]. Moreover, engagement with other legal or illegal substances, apart from alcohol, correlates with increased risk behavior. Creamer et al [10] demonstrated that using various tobacco products corresponds to heightened risk behavior. Additionally, a correlation exists between cannabis use, drinking-related risk behavior [11], and the frequency of alcohol-related consequences [12].

Given the known drinking motives among adolescents, prevention programs anchored in concepts like inoculation theory [13] and social learning theory [14] advocate for skill enhancement that strengthens resilience against social influences, such as peer pressure for risky behavior. Research involving life skills training in educational institutions underscores the value of cultivating social resistance skills and broader personal and social competencies, leading to decreased cigarette use and enhanced anti-drinking attitudes [15]. Moreover, participants demonstrated higher substance use awareness and skill-related knowledge [15]. Effective refusal communication skills and risk-related knowledge are crucial components for enhancing the personal and social skills of young people. Refusal communication, which involves the ability to say “no” to substance offers, is essential in reducing substance use and risky behavior. Several studies have demonstrated that individuals with strong refusal skills regarding alcohol misuse exhibit reduced abusive alcohol use [16-18], possess greater knowledge of alcohol misuse prevention, are less susceptible to peer pressure, and have better internal health control [19]. However, in addition to communication skills, it is also crucial for young people to have knowledge about substance use and its effects on the body to assess and avoid risks. Individuals with greater knowledge of substance-related topics may be better equipped to handle risky situations, potentially reducing the likelihood of substance abuse. Various studies have explored the relationship between substance-related knowledge and the reduction of risk behaviors, such as alcohol consumption. Teesson et al [20] conducted a cluster-randomized study in schools and found that combining digital prevention programs that increased alcohol-related knowledge resulted in a reduction in binge drinking. Hasking and Schofield [21] demonstrated that health and alcohol knowledge can strengthen the intention-behavior relationship. Individuals with more alcohol-related knowledge and experience are likely to be better informed about the consequences. Conversely, better-informed adolescents are likely to feel better prepared to minimize the risks of alcohol consumption [21]. Padget et al [22] discovered that increased awareness of the detrimental effects of alcohol on the brain resulted in improved perceptions of harm and subsequent attitudes of alcohol aversion. These improvements had a significant impact on the intention not to use alcohol, but they did not result in a significant reduction in short-term alcohol consumption. Therefore, it is important to note that knowledge about alcohol may be only one of many factors that can influence risk-taking decisions. Risk behaviors are frequently caused by multiple factors, and knowledge about alcohol may only have a partial impact on the development or absence of such behaviors.

Although traditional skills training involving rational alcohol consumption often relies on labor-intensive and costly in-person role-playing, contemporary technological solutions such as virtual reality (VR) have emerged. An intrinsic advantage of VR lies in its capacity to deliver an immersive encounter that faithfully mimics real-life scenarios. By replicating authentic situations, VR prompts participants to enact genuine behaviors within virtual environments [23,24]. Whereas studying real-world behaviors within experimentally controlled settings posed challenges, VR now allows one to scrutinize behaviors within genuine settings and uncover determinants of behavioral intentions [25].

The convergence between VR and real-life behavior might be attributed to presence and immersion. Individuals immersed in VR experiences can subjectively experience a sense of “being there” in the virtual realm. This sense of presence fosters more authentic and realistic behavioral responses akin to real-world conduct [26]. Alcañiz et al [27] reported that comparable neural mechanisms can be triggered in individuals immersed in a virtual world, paralleling experiences in the physical world. Additionally, the increasing realism and interactivity of VR technology play a role. As VR systems advance, they provide heightened sensory input and feedback, including lifelike visuals, haptic responses, and precise motion tracking. These immersive, lifelike elements contribute to greater congruence between VR and real-life behaviors [28]. Moreover, psychological factors such as social presence and adherence to social norms influence VR behavior, as people replicate real-world behaviors due to a sense of social presence and the desire to conform, even within virtual contexts [29].

The immersive, authentic portrayal of virtual environments implies that genuine risk behaviors are likely to manifest in these settings. This conjecture is supported by the findings that, for example, children exhibiting higher risk behavior in road traffic replicate this behavior in a virtual cycling simulation [30].

Nonetheless, reviews indicate that VR’s role in substance use prevention remains limited [31,32]. Thus, a cocreated virtual alcohol prevention simulation (VR FestLab) was developed in 2020 [33]. The VR FestLab application, an educational game simulation, aims to enhance the refusal self-efficacy of adolescents aged 15-18 years who experience social pressure to consume alcohol. Given the scarceness of VR-based alcohol prevention applications [31,32], this study was exploratory and aimed to ascertain if the established drinking motives and factors for risky drinking decisions are transferrable to VR FestLab. A structural equation model was used to uncover determinants linked to risky drinking decisions within the simulation. To this end, the following hypotheses were formulated for testing:

1. Higher sensation seeking is associated with increased virtual risk decisions in the simulation.
2. Enhanced knowledge and awareness of blood alcohol concentration (BAC), refusal communication skills, and drinking refusal skills are linked to reduced virtual risk decisions in the simulation.
3. Prior alcohol experiences are associated with increased virtual risk decisions in the simulation.

## Methods

### Study Design

The data were collected as part of a longitudinal study that investigated the efficacy of the VR FestLab application. The comprehensive procedure and outcomes of the primary study can be found elsewhere [34]. To achieve this objective, a total of 13 Danish schools were allocated in a 1:1 ratio to either the intervention or control group. The schools assigned to the intervention group engaged with the VR FestLab application, whereas those in the control group played the VR game First Steps (Meta Platforms Inc). The data set in this study was derived from the 7 schools that were selected as intervention schools only. Data collection transpired within the school premises between August 2020 and December 2020 and then again from April 2021 to May 2021; the latter period was necessitated by COVID-19 restrictions. For the original study [34], a sample size calculation was performed using STATA 15 with a 2-sample *t* test. This resulted in a sample size of 135 individuals for the control group and 135 individuals for the intervention group to yield an intervention effect of Cohen *d*=0.44, with a power of 0.80 using a 2-sided α of .05. The sample size was calculated based on an estimated intraclass correlation for drinking refusal self-efficacy of 0.01 and 45 students per school. Taking into account an estimated attrition of 35%, 420 participants were planned to be recruited for the study. Further information can be found elsewhere [34].

### Participants

To enroll adolescents aged between 14 and 18 years, initial contact was established with the administrations of the 7 schools through email. Once the school administrations granted their approval, the study’s objective was presented to the respective classes, emphasizing the confidential and anonymous nature of data collection. In accordance with the principles outlined in the Declaration of Helsinki, all students were duly informed that their participation was voluntary, and they gave written consent prior to their involvement.

### Ethical Considerations

The study adhered to Danish standards for the ethical conduct of scientific studies and was approved by the Research Ethics Committee of the University of Southern Denmark in March 2020 (case no 20/5348; date of approval: January 22, 2020). In accordance with the Ethics Committee of the University of Southern Denmark, parental consent was waived, as it is only mandatory for adolescents under the age of 15 years in Denmark [35].

### The Virtual Reality Simulation VR FestLab

**Figure 1:**
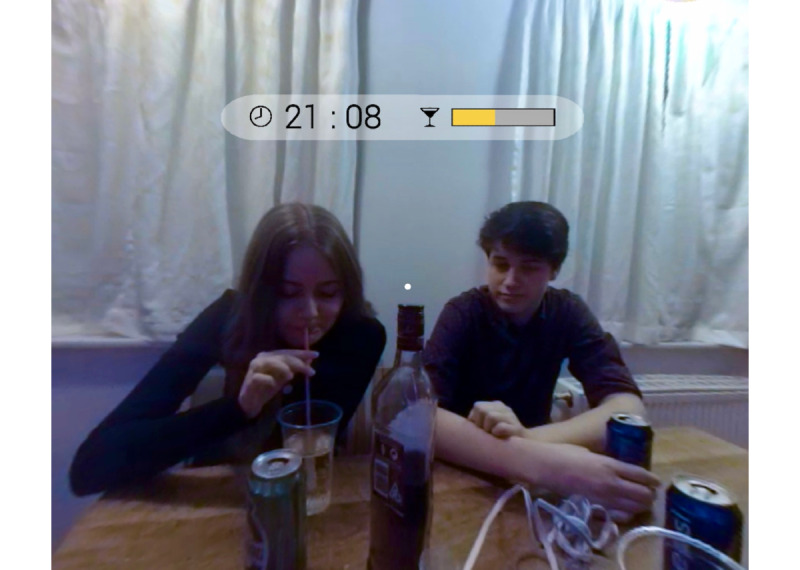
Screenshot of a scene from the virtual reality simulation FestLab.

The Danish VR application, VR FestLab, immerses users in a 360-degree filmed simulation and was specifically designed for adolescents aged between 15 and 18 years. A total of 128 distinct scenes allows users to engage with diverse simulation sequences. This interactive experience commences at the home of a school friend, where they both join in a birthday celebration. Within this simulation, users can navigate through 2 virtual rooms using eye movements. As they move within these spaces, they encounter various scenes, such as engaging in beer pong or participating in a flirting scenario, wherein they are presented with choices of both alcoholic and nonalcoholic beverages. Adhering to the taxonomy outlined in the Behavior Change Wheel [36], the simulation effectively integrates various behavior change functions, including education, training, modeling, and coercion or incentivization. Notably, individuals within the simulation can interact with role models who demonstrate the refusal to consume alcohol. If the user decides to consume alcohol and accepts an alcoholic drinking choice, the choice results in an incremental increase of a BAC bar, calculated via an algorithm, which is filled in at the top of the screen (see Figure 1, yellow BAC bar at the top of the screen).

This algorithm considers factors like gender, alcohol content in grams, drinking pace, and the average weight of a 16-year-old boy or girl to calculate the BAC [37]. To calculate the exact BAC score, a BAC calculation by Becker and Nielsen [38] was used. The calculation for girls and boys is as follows:



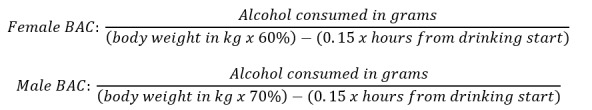



Should the user consume an excessive amount of alcohol within a short span, as determined by the algorithm, they suffer a blackout. This blackout is first portrayed by the camera shaking and a magnifying display of the BAC bar. Following this, within the game, the screen turns black, with the user subsequently awakening in a bedroom. In this bedroom, they receive messages that they have blacked out, concluding the simulation.

VR FestLab was pre-installed on Oculus Quest (Meta Platforms Inc) VR devices and handed out to the participants. Before engaging with the simulation, adolescents received instructions on device operation and navigation within the simulation. Following this, adolescents experienced VR FestLab for a maximum of 15 minutes in a classroom session. Depending on the simulation decisions, several rounds could be played during this time. After that, a structured 45-minute group reflection period was moderated by a trained study assistant in the classroom.

### Measures

Data were gathered through electronic questionnaires administered during school hours within classroom settings. The questionnaire was developed using the English versions of the respective scales (sensation seeking, drinking refusal skills, refusal communication skills) because no Danish versions were available (Multimedia Appendix 1). For the other measures (alcohol consumption, other drug consumption, knowledge and awareness of BAC) without existing scales in the literature, the project team created their own questions and items. The English questionnaires were translated to Danish and pretested with 31 students to determine their psychometric characteristics.

Adolescents were tasked with completing the questionnaire before (T0) and after (T1) the intervention. Independent variables, including age, gender, alcohol consumption, other drug consumption, sensation seeking, drinking refusal skills, knowledge and awareness of BAC, and refusal communication skills, were assessed at T0 before the VR FestLab intervention. The dependent variable, virtual risk behavior, was surveyed at T1 following the intervention.

### Virtual Risk Decisions

The primary outcome was virtual risk decisions, evaluated through a self-developed question: “Did you pass out at any time during the party?” Answer options were dichotomized (yes/no). Passing out can only be achieved in the simulation when the number and types of drinks accepted in a given time period resulted in a BAC of 2.0 permille, thus representing several risk decisions. The scenes and participant selections within the simulation were intentionally not recorded nor tracked to afford participants the utmost freedom in their interactions. As a result, the blackout experience in the game could only be assessed verbally. If participants responded affirmatively, it was inferred that the simulation terminated prematurely due to excessive alcohol consumption in a condensed time frame during the simulation.

### Age and Gender

Participant gender was dichotomously determined using the question “Are you a girl or a boy? (State what you most identify as right now),” while age was quantified using the question “How old are you?”

### Perceived Family Affluence, School Performance, and Health

To gather information on the sociodemographic factors of the participants, the following questions were adapted from the Health Behavior in School-aged Children study [39]: “How well-off do you think your family is?” “What does your class teacher(s) think about your school performance compared to your classmates?” and “Would you say your health is…?”

### Alcohol Consumption

Alcohol consumption was estimated with 3 single questions designed by the researchers. The questions “Have you ever drunk alcohol?” “Have you ever been drunk?” and ”Have you ever had 5 or more drinks on a single occasion?” could be answered dichotomously (yes/no) by the participants.

### Other Drug Consumption

To assess the consumption of substances other than alcohol, 5 customized questions were used. Participants were queried about their usage of cigarettes, hookah, e-cigarettes, snuff, or cannabis. Response options were dichotomized (yes/no).

### Sensation Seeking

Sensation seeking was measured using the 8-item Brief Sensation Seeking Scale (BSSS-8) by Hoyle et al [40], which has a Cronbach *α* of 0.76. Participants answered 8 statements using a 5-point Likert scale, ranging from “strongly disagree” to “strongly agree.” The 8-item scale contains 4 subscales with 2 items each. Subscales, including “thrill and adventure seeking,” “experience seeking,” “disinhibition,” and “boredom susceptibility,” were calculated following the criteria outlined by Hoyle et al [40]. The Cronbach *α* of the BSSS-8 was 0.76 in our sample.

### Drinking Refusal Skills—Social Pressure Subscale

To evaluate drinking refusal skills within the context of peer pressure, the 5-item social pressure subscale of the Drinking Refusal Self-Efficacy Questionnaire (DRSEQ) by Young et al [41], with a Cronbach *α* of 0.87, was used. Participants responded on a 6-point Likert scale, ranging from “I am very sure I could not resist drinking” to “I am very sure I could resist drinking.” The Cronbach *α* of the DRSEQ social pressure subscale was 0.88 in our sample.

### Knowledge and Awareness of Blood Alcohol Concentration

Knowledge and awareness of BAC was measured using 2 self-constructed items. Participants were prompted to rate the statements “It is easy for me to estimate my own alcohol tolerance” and “I know how much alcohol I can drink before I get drunk” on a 5-point Likert scale, spanning from “strongly disagree” to “strongly agree.”

### Refusal Communication Skills

Refusal communication skills were assessed using 2 items drawn from the Alcohol Misuse Prevention Knowledge Questionnaire by Shope et al [19]. Only 2 items from the questionnaire by Shope et al [19] were used because the other questionnaire items do not cover relevant aspects of VR FestLab [34]. Participants were asked to evaluate the statements “If someone offers me a drink of alcohol and I say no, I can make them take no for an answer” and “If my best friends want me to drink beer with them and I don’t want to, I have ways to say no” on a 5-point Likert scale, ranging from “strongly disagree” to “strongly agree.”

### Statistical Analysis

Data analysis was performed using the R Studio software package (version 2022.07.2). The 2-step methodology, as outlined by Herting and Costner [42], was used to calculate model fit as the first step and formulate the structural equation model as the second step. Initially, confirmatory factor analysis (CFA) using the lavaan package [43] was carried out to determine the adequacy of the measurement models of the questionnaires. For this purpose, different single-factor models were created and calculated using a diagonally weighted least squares estimator that is suitable for categorical items. Model fit was checked using a chi-square test in combination with other fit indices such as the comparative fit index (CFI≥0.95), root mean square error of approximation (RMSEA≤0.06), Tucker-Lewis index (≥0.95), and standardized root mean square residual (SRMR≤0.08) [44].

Subsequently, a structural model with latent variables was calculated to investigate the influence of the constructs (alcohol consumption, other drug consumption, sensation seeking, drinking refusal skills, knowledge and awareness of BAC, and refusal communication skills) on the main outcome (virtual risk decisions) and the correlation of the constructs with age and gender as covariates. In addition, a moderation analysis for gender and age was run. Estimated scores for each latent variable were calculated and subsequently incorporated into a logistic regression model to predict virtual risk decisions.

## Results

### Participants

A total of 268 students from 7 schools were assigned to the intervention group. Of these, 183 students participated in the baseline survey (T0). The main reasons for dropout between allocation and baseline survey were that 1 complete school (n=36) dropped out, 1 complete school class (n=15) dropped out, and 34 students were not willing to participate or did not provide consent. After the intervention, 2 additional participants dropped out, resulting in 181 individuals completing the first follow-up questionnaire (T1). The subsequent analysis was based on a complete data set from 161 participants. Additional information on allocation and dropouts can be found elsewhere [34]. To test whether there were differences between respondents at T1 and those who provided complete information at T1 and were included in the analysis, independent *t* tests were conducted for metric variables, and chi-square tests were conducted for nominal and ordinal scaled sociodemographic variables. The analyses showed no differences between the 2 groups in terms of age (*t*_179_=0.61, *P*=.54), gender (*χ*^2^_1_=0.31, *P*=.58), and Family Affluence Scale (FAS; *χ*^2^_1_=0.25, *P*=.42). For the chi-square analysis of the FAS between completers and noncompleters, the prerequisite of cell frequencies above 5 was violated, which is why a Fisher exact test was used.

### Sample Characteristics

Table 1 provides an overview of the demographic attributes. Gender distribution was equal, with 78 of the 161 (48.4%) participants being female. The mean age of the sample was 15.6 (SD 0.72) years. Most respondents (143/161, 88.8%) reported low to moderate perceived family affluence, and 62.1% (100/161) rated their perceived school performance as good to very good. Additionally, 79.5% (128/161) reported good to very good health. The majority of respondents (119/161, 73.9%) reported having engaged in binge drinking at some point in their lives.

**Table 1:** Characteristics of the study population (n=161).

Characteristics			Value
Age (years), mean (SD)			15.6 (0.72)
Gender (female), n (%)			78 (48.4)
**Perceived family affluence, n (%)**			
	Low to medium	143 (88.8)	
	High to very high	18 (11.2)	
**Perceived school performance, n (%)**			
	Good to very good	100 (62.1)	
	Below average to average	61 (37.9)	
**Perceived health and well-being, n (%)**			
	Good to excellent	128 (79.5)	
	Poor to fair	33 (20.5)	
**Lifetime binge drinking, n (%)**			
	Yes	119 (73.9)	
	No	42 (26.1)	

### Calculations of Model Fit

**Figure 2:**
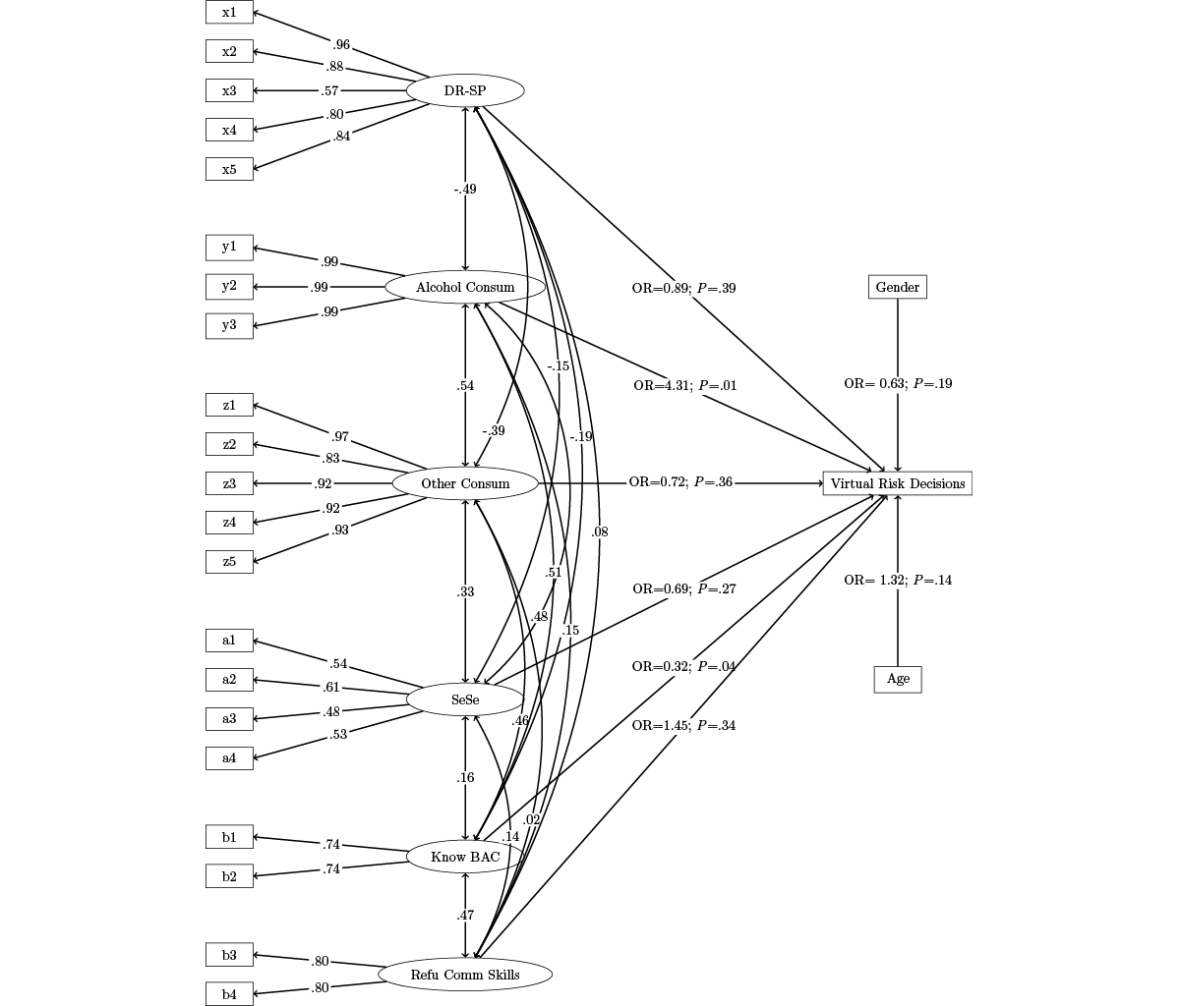
Path model of the relationships between virtual risk decisions and drinking-refusal skills-social pressure (DR-SP), alcohol consumption (Alcohol Consum), other drug consumption (Other Consum), sensation seeking (SeSe), knowledge and awareness of blood alcohol concentration (Know BAC), and refusal communication skills (Refu Comm Skills). OR: odds ratio.

The single-factor models generally showed a good fit in the CFA and could therefore be transferred to the structural equation model without adjustments. Sensation seeking provided a mediocre fit, with CFI and RMSEA beyond their cut-offs, while the 90% CI of the RMSEA included the cut-off (see Table 2). The factors “knowledge and awareness of BAC” and “refusal communication skills” were combined for the CFA for statistical reasons; otherwise, it would not have been possible to determine the fit, as they only contained 2 items each. Finally, the combination of the 2 factors showed high loadings on the individual and superordinate factors, which resulted in good fit values in the CFA. The results of the single-factor models are shown in Table 2 and Figure 2.

**Table 2:** The goodness of fit indices of the confirmatory factor analysis for single-factor models.

Model	Items	*χ*^2^ test (*df*)	*P* value	CFI^a^	TLI^b^	SRMR^c^	RMSEA^d^ (90% CI)	*P* value
Alcohol consumption	3	2.24 (2)	.33	1.00	1.00	0.04	0.03 (0.00-0.16	.46
Other drug consumption	5	3.02 (5)	.70	1.00	1.00	0.02	0.00 (0.00-0.08)	.84
Sensation seeking	4	7.38 (2)	.03	0.92	0.76	0.05	0.13 (0.04-0.23)	.07
Drinking refusal skills: social pressure subscale	5	19.47 (5)	.01	0.97	0.95	0.03	0.13 (0.08-0.20)	.01
Knowledge of BAC^e^ and refusal communication skills	4	1.42 (1)	.23	1.00	0.99	0.02	0.05 (0.00-0.22)	.32

**^a^**CFI: comparative fit index.

^b^TLI: Tucker-Lewis index.

^c^SRMR: standardized root mean square residual.

^d^RMSEA: root mean square error of approximation.

^e^BAC: blood alcohol concentration.

### Determinants Linked to Risky Drinking Decisions

A general overview of the bivariate correlations of all variables used in this study can be found in Multimedia Appendix 2. Age and gender were included as covariates without moderation in the structural equation model, as shown in Figure 2. The path model used to test the formulated hypotheses showed an unsatisfying model fit: *χ*^2^_238,161_=422.59, *P*<.001; CFI=0.94, SRMR=0.12, RMSEA=0.07 (90% CI 0.06-0.08). Nevertheless, we decided to retain the model, because all included measurement models were a priori tested and showed good fit. Due to the explorative nature of the study, the structural model included all possible paths. Therefore, the reason for misfit can be attributed to the cross-loadings of various scales only. As the structural part of the model was the aim of the study, we decided to retain it. Figure 2 shows the tested model. Prior alcohol experiences and knowledge and awareness of BAC were significantly associated with virtual risk decisions in VR FestLab. Participants who reported prior alcohol consumption had a 4.31-fold higher chance of showing virtual risk decisions in the simulation (odds ratio [OR] 4.31, 95% CI 1.70-10.84; *P*=.01). Therefore, hypothesis 3 could be accepted. Apart from that, adolescents with high knowledge and awareness of BAC at baseline were 0.32 times less likely to make virtual risk decisions in the simulation (OR 0.32, 95% CI 0.11-0.93; *P*=.04). Higher social pressure drinking refusal skills (OR 0.89, 95% CI 0.69-1.16; *P*=.39), consumption of other drugs (OR 0.72, 95% CI 0.36-1.45; *P*=.36), sensation seeking (OR 0.69, 95% CI 0.36-1.34; *P*=.27), or refusal communication skills (OR 1.45, 95% CI 0.68-3.10; *P*=.34) showed no significant relationship with virtual risk decisions in the simulation. Therefore, hypotheses 1 had to be rejected, and hypothesis 2 could only be partially confirmed.

### Moderation Effects of Gender or Age Regarding Virtual Risk Decisions

Subsequently, we analyzed whether age and gender had a moderating influence on the latent variables. The results of this moderation analysis are presented in Multimedia Appendix 3. All individual moderation analyses for the variables (alcohol consumption, other drug consumption, sensation seeking, drinking refusal skills, knowledge and awareness of BAC, and refusal communication skills) showed no significant moderation. Accordingly, age and gender did not significantly alter the effects of the tested constructs on virtual risk decisions in the simulation. Age and gender also did not significantly influence the dependent variable of virtual risk decisions (Multimedia Appendix 3).

## Discussion

### Principal Findings

In summary, the structural equation model exhibited a significant association between prior alcohol experiences and knowledge and awareness of BAC with virtual risk decisions, supporting hypothesis 3. The other initially hypothesized factors (hypotheses 1 and 2), including drinking refusal skills, sensation seeking, refusal communication skills, and consumption of other drugs, did not have significant associations with virtual risk decisions. Upon exploring moderating factors such as age and gender, no moderation on virtual risk decisions was identified. This study has underscored that prior alcohol experiences are notably linked to virtual risk decisions. Participants who have encountered alcohol and engaged in binge drinking appear to perceive the VR FestLab game as realistically simulating their personal behaviors, leading them to enact these behaviors within the game. Apart from that, it appears that higher knowledge and awareness of BAC is a protective factor and leads to fewer virtual risk decisions in the simulation.

### Comparison With Prior Work

These findings align with qualitative insights collected from focus group investigations involving adolescents discussing the simulation [45,46]. Adolescents reported finding the VR simulation remarkably realistic, evoking sensations akin to being present at an actual party [45,46]. The initial participation in VR FestLab possibly aimed to ascertain whether the simulated party aligned with their expectations and whether outcomes matched real-world drinking behaviors. The study’s data set does not offer insights into potential variations in behavior between several attempts with VR FestLab. However, it seems plausible that distinct behaviors and strategies would be attempted in subsequent trials, as highlighted by focus group participants in qualitative interviews. The participants expressed the view that VR serves as a medium for experimenting with various behaviors and that repeated engagements with the VR simulation allow for exploring different strategies, such as drinking versus abstaining, while observing the reactions of simulation characters [46]. This points to VR’s potential benefit in alcohol prevention, allowing participants to experiment with diverse approaches and behaviors in risky scenarios. This study found a relatively high lifetime prevalence of binge drinking in the group of adolescents aged 14 years to 18 years, at about 73.9% (119/161). In this context, it would be interesting for future studies to investigate the association between previous alcohol consumption and the likelihood of risk decisions in different social environments. These results may also provide hints for further virtual scenarios that can be integrated into the VR FestLab. Hadley et al [47], who combined VR environments with emotion regulation and a risk reduction intervention, also arrived at similar conclusions, indicating that VR, through better simulated contextual cues of risky situations, facilitates the application of different emotion regulation strategies among adolescents. In the real world, such experimentation of behavioral strategies is constrained by the necessity of personally experiencing risky behavior and its consequences, often without the possibility of multiple trials (eg, in the case of blackout).

The hypothesis that individuals with high sensation-seeking tendencies would exhibit elevated virtual risk decisions within the game could not be substantiated by the study’s findings. The lack of a significant association between sensation seeking and virtual risk decisions could potentially be attributed to the design of VR FestLab. The game’s preventive nature might not have resonated sufficiently with individuals displaying high sensation-seeking behavior. Given that sensation seeking characterizes those seeking diverse, potentially risky experiences [48], the simulated risk behavior in VR FestLab might not have provided compelling cues for such participants. Participants were aware of the virtual and simulated nature of the risk decisions, possibly leading to the observed absence of virtual risk decisions in the game. This absence could also stem from the absence of personal consequences, such as intoxication, emotional arousal, and sensory perceptions, conveyed through VR FestLab. This lack of stimuli might not engage adolescents with a high sensation-seeking drive. On the contrary, sensation seeking is a multifaceted construct, not exclusively tied to risky behavior. The study by Ravert and Donnellan [49] found that sensation seeking, manifested as a search for stimulation, could also be positively linked to psychological well-being. Furthermore, sensation seeking might have been manifest in nonalcohol-related scenes within VR FestLab (eg, interactions with game characters), rendering the presumed virtual risk decisions unappealing to participants with pronounced sensation-seeking tendencies.

Likewise, drinking refusal self-efficacy skills failed to display an association with reduced virtual risk decisions in this study. Those with higher baseline drinking refusal self-efficacy skills might have opted for less risky drinking decisions within VR FestLab, possibly bypassing activities like beer pong. These observations align with those of the study by Guldager et al [34] that assessed the efficacy of VR FestLab. Although effects were insignificant, a more pronounced increase in drinking refusal skills was noted among participants whose baseline skills lay below the median compared with those above the median. Despite the documented real-world associations between risky behavior and higher drinking refusal skills, drug consumption, and refusal communication skills [50,51], these correlations did not manifest in the virtual world.

Although it was originally hypothesized that higher refusal communication skills would be associated with a reduction in virtual risk decisions, this hypothesis was not supported by this study. It is possible that participants were more exploratory when playing VR FestLab to “just see what happens next” and did not use their refusal communication skills in the simulation to say no to drinking offers. Qualitative interviews with adolescents who played VR FestLab revealed that they felt the peer pressure in the simulation was weaker than in real life [45], which could be a reason why refusal communication skills were not associated with reduced virtual risk decisions in this study.

Apart from that, this study has shown that individuals with higher knowledge and awareness of BAC are less likely to make risky decisions in virtual environments. It is reasonable to assume that those who understand how different drinks affect BAC are less likely to engage in risky decisions in VR FestLab compared with those who cannot accurately assess the effects of BAC. These assumptions are supported by the literature, which indicates that individuals with greater knowledge of low-risk alcohol consumption are more likely to reduce their alcohol intake and make fewer risky decisions [52]. Increased awareness of BAC could enhance risk perception and enable better anticipation and assessment of the consequences of risk decisions. It is possible that individuals without knowledge and awareness of BAC were more willing to experiment in the simulation, leading to an increase in virtual risk-taking decisions. VR FestLab is a prevention program that uses an in-game BAC bar to illustrate the effects of alcohol-related decisions on BAC. The in-game presentation may help adolescents avoid risky decisions by increasing their knowledge of BAC. Other research [53] using VR in e-cigarette prevention has also shown that virtual prevention simulations can increase substance-related knowledge and harmful perceptions about e-cigarettes.

To our knowledge, this study is the first attempt to examine the relationship between real-life behavior and attitudes and virtual risk decisions in the context of alcohol prevention, making it difficult to contextualize the findings with those of other virtual alcohol prevention studies. The findings illuminate VR’s capacity to replicate real-life behavior within simulated environments. Other studies [54-56] using VR in other thematic areas have also concluded that there are links between simulated behavior and real behavior. The combination of prevention and VR within research is relatively novel, necessitating further exploration into leveraging simulated settings for risk behavior prevention and health promotion. Existing studies underscore increasing adoption of VR for prevention and health promotion [57]. Building behavior change interventions and predicting behavioral transformations are challenging in real-world scenarios [58]. Immersive technologies, such as VR, can harness specific attributes like training and realism to facilitate future behavioral change. Nevertheless, the translation of real-life behavior into virtual simulations, along with the potential impact of virtually acquired behavioral patterns on subsequent real-world behavior, remains uncertain. Research suggests that altering an individual’s avatar representation in VR influences their behavior and psychological disposition in the real world, an occurrence termed the Proteus effect [24,59]. This phenomenon could also be probed in the context of alcohol prevention, where avatars could shape participants’ self-image and preventive behaviors. Addressing these unresolved questions in future studies while delving into the mechanisms at play in virtual simulations could position VR as a valuable asset in prevention and health promotion.

### Limitations

In this study, certain limitations must be acknowledged when interpreting the results. First, the applicability of the findings warrants consideration. Participants were drawn from diverse Danish schools, making the results pertinent exclusively to Danish students. Additionally, the recruitment was limited to a specific region within Denmark, implying that adolescents from other regions within Denmark or other countries might exhibit disparate outcomes.

Second, the results should be cautiously approached due to the reliance on self-reported data, which are susceptible to common biases such as respondent bias, extreme responding, and social desirability bias [60]. These potential biases could have influenced and skewed the reported results. The timing of the follow-up data collection (T1), conducted immediately after the intervention, may have influenced the results. Furthermore, this study did not survey risk behaviors that may occur prior to a blackout, such as engaging in sexual activity or driving under the influence of alcohol. Future studies should consider measuring these risk behaviors before a blackout occurs in order to establish a relationship. Specifically, the question about certain risk decisions may have been biased (eg, experiencing a blackout). The potential for a Hawthorne effect should be considered when interpreting the results, because the students in the intervention group were not blinded and tested the new VR FestLab application. Their knowledge of participation in the study and the aim of VR FestLab can influence their self-assessment of whether they experienced a blackout in the application, which could potentially distort the results. Since there was no content tracking during participants’ engagement with the simulation, the navigation patterns within the simulation remain uncharted. Integrating a content log in future analyses could offer deeper insights into participants’ usage behavior.

Third, organizational limitations limited the playing time to 15 minutes for each participant. This restriction could have led to variations in the number of rounds played within the simulation, potentially affecting the reported outcomes. Additionally, the controlled setting of the VR FestLab intervention study might have curtailed the expression of extreme behaviors that could naturally occur among adolescents. This controlled environment may have influenced the observed results.

### Conclusions

Despite these limitations, this study illuminates the link between real-life behavior and virtual simulations in alcohol prevention. The significance of prior alcohol experiences in shaping virtual risk decisions underscores the potential of VR to mimic real behaviors. The study highlights VR’s potential as a valuable tool for behavior change interventions and health promotion. To harness this potential, future research should delve into mechanisms underpinning behavioral expression in virtual environments. As VR evolves, it presents a promising avenue for health promotion strategies, offering a unique medium to experiment with behaviors in risk-prone scenarios. This research signifies the importance of advancing our understanding of the intersection between human behavior, virtual simulations, and their real-world implications.

## Appendix

Multimedia Appendix 1 Investigator-developed questionnaire.

Multimedia Appendix 2 Bivariate correlations of all variables.

Multimedia Appendix 3 Results of the moderation analyses.

## Abbreviations

BAC: blood alcohol concentration
BSSS: Brief Sensation Seeking Scale
CFA: confirmatory factor analysis
CFI: comparative fit index
DRSEQ: Drinking Refusal Self-Efficacy Questionnaire
FAS: Family Affluence Scale
OR: odds ratio
RMSEA: root mean square error of approximation
SRMR: standardized root mean square residual
TLI: Tucker-Lewis index
VR: virtual reality

## Data Availability

The analyzed data can be accessed from the Zenodo database [61].
