# Supplementary material for: Factors Associated With Risky Drinking Decisions in a Virtual Reality Alcohol Prevention Simulation: Structural Equation Model
Source: JMIR XR Spat Comput. 2024 May 6;1:e56188. doi: 10.2196/56188 (PMC12671314; doi:10.2196/56188)
Supplement: Multimedia Appendix 1 [file xr_v1i1e56188_app1.pdf]

Sociodemographic

1. What class are you in?

(1) ❑ 9th grade

(2) ❑ 10th grade.

2. How old are you?

(1) ❑ 14

(2) ❑ 15

(3) ❑ 16

(4) ❑ 17

(5) ❑ 18

(6) ❑ 19

3. Are you a girl or a boy?

(1) ❑ Boy

(2) ❑ Girl

4. How well off do you think your family is?

(1) ❑ Very well off

(2) ❑ Quite well off

(3) ❑ Average

(4) ❑ Not so well off

(5) ❑ Not at all well off

School performance

5. In your opinion, what does your class teacher(s) think about your school performance compared to your classmates?

(1) ❑ Very good

(2) ❑ Good

(3) ❑ Average

(4) ❑ Below average

Health and wellbeing

6. Would you say your health is……?

(1) ❑ Excellent

(2) ❑ Good

(3) ❑ Fair

(4) ❑ Poor

Alcohol and other drug consumption

8. Have you ever used any of the following substances?

	Yes	No
a) Water pipe	(1) ❑	(2) ❑
b) E-cigarettes	(1) ❑	(2) ❑
c) Moist snuff (snus)	(1) ❑	(2) ❑
d) Cannabis or marijuana	(1) ❑	(2) ❑
e) Tobacco	(1) ❑	(2) ❑

9. Have you ever…

	**Yes**	**No**
Been drinking alcohol?	(1) ❑	(2) ❑
Been drunk? (been drinking so much, that you either are very happy, sad, dizzy or tired)	(1) ❑	(2) ❑
Been drinking 5 or more drinks at the same occasions?	(1) ❑	(2) ❑

Drinking Refusal skills – social pressure subscale

10. How sure are you that you could resist drinking alcohol?

	I am very sure I could NOT resist drinking	I most likely could NOT resist drinking	I probably could NOT resist drinking	I probably could resist drinking	I most likely could resist drinking	I am very sure I could resist drinking
When I am at a party	(1) ❑	(2) ❑	(3) ❑	(4) ❑	(5) ❑	(6) ❑
When someone offers me a drink	(1) ❑	(2) ❑	(3) ❑	(4) ❑	(5) ❑	(6) ❑
When a boy/girl I like is drinking	(1) ❑	(2) ❑	(3) ❑	(4) ❑	(5) ❑	(6) ❑
When my friends are drinking	(1) ❑	(2) ❑	(3) ❑	(4) ❑	(5) ❑	(6) ❑
When I am going out with my friends	(1) ❑	(2) ❑	(3) ❑	(4) ❑	(5) ❑	(6) ❑

Communication skills

11. Answer according to how much you agree or disagree with each statement.

	Disagree strongly	Disagree somewhat	Uncertain	Agree somewhat	Agree strongly
If your best friends want you to drink beer with them and you don`t want to, do you have ways to say no?	(1) ❑	(2) ❑	(3) ❑	(4) ❑	(5) ❑
If someone offers you a drink of alcohol and you say “no”, can you make them take “no” for an answer?	(1) ❑	(2) ❑	(3) ❑	(4) ❑	(5) ❑

Knowledge of alcohol consumption

12. Answer according to how much you agree or disagree with each statement.

	Disagree strongly	Disagree somewhat	Uncertain	Agree somewhat	Agree strongly
It is easy for me to estimate my own alcohol tolerance.	(1) ❑	(2) ❑	(3) ❑	(4) ❑	(5) ❑
I know how much alcohol I can drink before I get drunk	(1) ❑	(2) ❑	(3) ❑	(4) ❑	(5) ❑

Sensation seeking

13. In the following we ask you some questions about how you explore new things in life.

	Disagree strongly	Disagree somewhat	Uncertain	Agree somewhat	Agree strongly
I would like to explore strange places.	(1) ❑	(2) ❑	(3) ❑	(4) ❑	(5) ❑
I would like to take off on a trip with no pre-planned routes or timeta-bles.	(1) ❑	(2) ❑	(3) ❑	(4) ❑	(5) ❑
I get restless when I spend too much time at home.	(1) ❑	(2) ❑	(3) ❑	(4) ❑	(5) ❑
I prefer friends who are excitingly unpredictable.	(1) ❑	(2) ❑	(3) ❑	(4) ❑	(5) ❑
I like to do frightening things.	(1) ❑	(2) ❑	(3) ❑	(4) ❑	(5) ❑
I would like to try bungee jumping.	(1) ❑	(2) ❑	(3) ❑	(4) ❑	(5) ❑
I like wild parties.	(1) ❑	(2) ❑	(3) ❑	(4) ❑	(5) ❑
I would love to have new and excit-ing experiences, even if they are ille-gal.	(1) ❑	(2) ❑	(3) ❑	(4) ❑	(5) ❑
