# Supplementary material for: Factors Associated With Risky Drinking Decisions in a Virtual Reality Alcohol Prevention Simulation: Structural Equation Model
Source: JMIR XR Spat Comput. 2024 May 6;1:e56188. doi: 10.2196/56188 (PMC12671314; doi:10.2196/56188)
Supplement: Multimedia Appendix 2 [file xr_v1i1e56188_app2.pdf]

Multimedia Appendix 2. Bivariate correlations of all variables.

Measures	1	2	3	4	5	6	7	8	9	10	11	12	13	14	15	16	17	18	19	20	21
1. Alcohol consumption	–																				
2. Drunkenness	1.00	–																			
3. Binge drinking	1.00	0.98	–																		
4. Cigarettes consumption	0.55	0.55	0.51	–																	
5. Water pipes consumption	0.13	0.21	0.15	0.86	–																
6. E-cigarettes consumption	0.49	0.54	0.50	0.90	1.00	–															
7. Snus consumption	0.94	0.72	0.62	0.87	0.75	0.86	–														
8. Hash consumption	0.90	0.38	0.55	0.86	0.79	0.86	0.84	–													
9. Sensation seeking disinhibition	0.22	0.21	0.16	-0.09	-0.09	-0.10	-0.13	-0.01	–												
10. Sensation seeking experience	0.08	0.27	0.22	0.13	0.14	0.22	0.20	0.23	0.31	–											
11. Sensation seeking boredom	0.11	0.18	0.08	0.01	0.03	0.08	0.07	0.11	0.40	0.24	–										
12. Sensation seeking thrill	0.41	0.52	0.50	0.47	0.35	0.44	0.49	0.50	0.19	0.32	0.37	–									
13. Drink refusal skills social pressure a	-0.38	-0.54	-0.60	-0.44	-0.26	-0.37	-0.33	-0.46	0.05	-0.17	0.09	-0.42	–								
14. Drink refusal skills social pressure b	-0.33	-0.53	-0.47	-0.41	-0.23	-0.31	-0.33	-0.41	0.09	-0.06	0.02	-0.40	0.70	–							
15. Drink refusal skills social pressure c	-0.12	-0.35	-0.22	-0.11	-0.24	-0.19	-0.08	-0.20	0.02	-0.10	0.02	-0.32	0.50	0.54	–						
16. Drink refusal skills social pressure d	-0.30	-0.45	-0.48	-0.27	-0.09	-0.23	-0.19	-0.36	0.04	-0.12	0.04	-0.38	0.77	0.68	0.66	–					
17. Drink refusal skills social pressure c	-0.28	-0.42	-0.40	-0.38	-0.23	-0.27	-0.32	-0.48	0.03	-0.14	0.02	-0.42	0.74	0.71	0.56	0.73	–				
18. Knowledge about alcohol tolerance	0.11	0.31	0.34	0.23	0.38	0.19	0.24	0.28	0.09	0.14	-0.01	-0.05	0.01	0.09	-0.07	0.01	-0.05	–			
19. Knowledge about drinking	0.32	0.49	0.51	0.46	0.36	0.25	0.35	0.28	0.05	0.07	0.02	0.17	-0.24	-0.23	-0.24	-0.25	-0.27	0.55	–		
20. Communication skills decline	-0.02	0.21	0.07	0.02	0.00	0.07	-0.01	-0.05	0.18	0.00	0.00	-0.24	0.11	0.11	0.19	0.18	0.00	0.36	0.23	–	
21. Communication skills self-assertion	0.09	0.16	0.20	0.00	-0.03	-0.03	-0.01	0.19	0.28	0.08	0.11	0.01	-0.01	-0.07	0.06	0.04	-0.00	0.26	0.25	0.63	–
22. Virtual risk decisions	0.33	0.48	0.51	0.04	-0.17	-0.00	0.12	0.02	-0.15	-0.02	-0.00	0.31	-0.31	-0.28	-0.19	-0.26	-0.20	-0.04	-0.09	-0.04	0.07
