# Supplementary material for: Factors Associated With Risky Drinking Decisions in a Virtual Reality Alcohol Prevention Simulation: Structural Equation Model
Source: JMIR XR Spat Comput. 2024 May 6;1:e56188. doi: 10.2196/56188 (PMC12671314; doi:10.2196/56188)
Supplement: Multimedia Appendix 3 [file xr_v1i1e56188_app3.pdf]

Multimedia Appendix 3. Results of the moderation analyses

Moderation between alcohol consumption and gender

	Estimate	Std.error	Z value	P-Value
Intercept	-0.50	0.24	-2.105	.03
Alcohol consumption	0.35	0.32	1.256	.27
Gender	-0.49	0.35	-1.360	.16
Alcohol consumption : Gender	0.63	0.50	1.296	.21

Moderation between other consumption and gender

	Estimate	Std.error	Z value	P-Value
Intercept	-0.53	0.24	-2.22	.03
Other consumption	0.05	0.34	0.14	.88
Gender	-0.46	0.35	-1.32	.19
Other consumption : Gender	0.21	0.48	0.44	.66

Moderation between sensation seeking and gender

	Estimate	Std.error	Z value	P-Value
Intercept	-0.52	0.23	-2.23	.02
Sensation seeking	-0.07	0.27	-0.25	.80
Gender	-0.44	0.34	-1.30	.19
Sensation seeking : Gender	0.46	0.40	1.15	.25

Moderation between drinking refusal skills and gender

	Estimate	Std.error	Z value	P-Value
Intercept	-0.54	0.24	-2.25	.02
Drinking refusal skills	-0.26	0.16	-1.59	.11
Gender	-0.56	0.36	-1.53	.13
Drinking refusal skills : Gender	-0.19	0.23	-0.82	.41

Moderation between knowledge/awareness of BAC and gender

	Estimate	Std.error	Z value	P-Value
Intercept	-0.52	0.24	-2.20	.03
Knowledge/awareness of BAC	-0.19	0.40	-0.48	.63
Gender	-0.43	0.34	-1.26	.21
Knowledge/awareness of BAC : Gender	0.41	0.57	0.72	.47

Moderation between refusal communication skills and gender

	Estimate	Std.error	Z value	P-Value
Intercept	-0.50	0.24	-2.11	.03
Refusal communication skills	-0.29	0.40	-0.71	.48
Gender	-0.41	0.35	-1.19	.24
Refusal communication skills : Gender	0.54	0.57	0.96	.34

Moderation between alcohol consumption and age

	Estimate	Std.error	Z value	P-Value
Intercept	-0.74	0.17	-4.20	<.01
Alcohol consumption	0.58	0.25	2.37	.02
Age	0.20	0.18	1.11	.27
Alcohol consumption : Age	0.03	0.24	0.12	.90

Moderation between other consumption and age

	Estimate	Std.error	Z value	P-Value
Intercept	-0.77	0.17	-4.42	<.01
Other consumption	0.19	0.24	0.80	.42
Age	0.26	0.18	1.47	.14
Other consumption : Age	0.11	0.26	0.42	.67

Moderation between sensation seeking and age

	Estimate	Std.error	Z value	P-Value
Intercept	-0.75	0.17	-4.40	<.01
Sensation seeking	0.14	0.20	0.70	.49
Age	0.25	0.17	1.45	.15
Sensation seeking : Age	0.12	0.22	0.54	.60

Moderation between drinking refusal skills and age

	Estimate	Std.error	Z value	P-Value
Intercept	-0.80	0.18	-4.49	<.01
Drinking refusal skills	-0.33	0.12	-2.90	<.01
Age	0.24	0.18	1.30	.19
Drinking refusal skills : Age	-0.01	0.12	-0.05	.96

Moderation between knowledge/awareness of BAC and age

	Estimate	Std.error	Z value	P-Value
Intercept	-0.76	0.17	-4.40	<.01
Knowledge/awareness of BAC	-0.02	0.29	-0.06	0.95
Age	0.27	0.18	1.51	0.13
Knowledge/awareness of BAC : Age	0.08	0.29	0.28	0.77

Moderation between refusal communication skills and age

	Estimate	Std.error	Z value	P-Value
Intercept	-0.75	0.17	-4.34	<.01
Refusal communication skills	0.04	0.28	0.14	0.89
Age	0.25	0.17	1.41	0.16
Refusal communication skills : Age	-0.06	0.30	-0.20	0.84

Moderation of age and gender on virtual risk behaviour and age

	Estimate	Std.error	Z value	P-Value
Intercept	-0.39	0.26	-147	.14
Alcohol consumption	1.83	0.80	2.30	.02
Other consumption	-0.39	0.42	-0.94	.35
Sensation seeking	-0.54	0.34	-1.58	.11
Drinking refusal skills	-0.13	0.19	-0.67	.50
Knowledge/awareness of BAC	-1.37	0.64	-2.15	.03
Refusal communication skills	0.41	0.42	0.98	.33
Age	0.15	0.19	0.78	.44
Gender	-0.61	0.37	-1.62	.11
